# Towards a fully functional integrated photonic-electronic platform via a single SiGe growth step

**DOI:** 10.1038/srep19425

**Published:** 2016-01-19

**Authors:** Callum G. Littlejohns, Thalia Dominguez Bucio, Milos Nedeljkovic, Hong Wang, Goran Z. Mashanovich, Graham T. Reed, Frederic Y. Gardes

**Affiliations:** 1 Optoelectronics Research Centre, Building 53, University of Southampton, Southampton, SO17 1BJ, UK; 2 Novitas, Nanoelectronics Centre of Excellence, Nanyang Technological University, 50 Nanyang Avenue, Singapore, 639798

## Abstract

Silicon-germanium (Si_1-x_Ge_x_) has become a material of great interest to the photonics and electronics industries due to its numerous interesting properties including higher carrier mobilities than Si, a tuneable lattice constant, and a tuneable bandgap. In previous work, we have demonstrated the ability to form localised areas of single crystal, uniform composition SiGe-on-insulator. Here we present a method of simultaneously growing several areas of SiGe-on-insulator on a single wafer, with the ability to tune the composition of each localised SiGe area, whilst retaining a uniform composition in that area. We use a rapid melt growth technique that comprises of only a single Ge growth step and a single anneal step. This innovative method is key in working towards a fully integrated photonic-electronic platform, enabling the simultaneous growth of multiple compositions of device grade SiGe for electro-absorption optical modulators operating at a range of wavelengths, photodetectors, and bipolar transistors, on the same wafer. This is achieved by modifying the structural design of the SiGe strips, without the need to modify the growth conditions, and by using low cost, low thermal-budget methods.

Silicon is widely regarded as the substrate of choice for future photonic devices and systems. It has the lowest cost and highest crystal quality of any semiconductor material^1^, and decades of fabrication knowledge and expertise have been established by the complementary metal-oxide semiconductor (CMOS) industry. Most conventional Si photonic devices^2^,^3^,^4^,^5^,^6^,^7^,^8^,^9^,^10^,^11^ are fabricated using silicon-on-insulator (SOI) substrates^12^ for optical confinement purposes. However, the migration of Si photonics to bulk Si substrates, with localised SiGe-on-insulator (SGOI) layers for active photonic and electronic devices^13^ (SiGe has higher carrier mobilities than Si^14^), is of major interest for cost, process flexibility, and electronic compatibility reasons. A CMOS compatible electronic-photonic integrated platform, on Si substrates, requires the growth of Ge for photodetection^15^,^16^. Additionally, the growth of SiGe for electro-absorption (EA) modulators^17^ is desirable. The EA modulator has unique advantages for use in electronic–photonic integration because it forms devices on the micrometre scale, as opposed to the millimetre scale of other modulator designs such as Si Mach-Zehnder based designs, whilst also demonstrating a very low energy consumption of 10–100 fJ per bit^18^. The EA effect is an ultrafast process that takes place in sub-picosecond timescales^19^, and in principle, is therefore capable of >100 GHz high-speed modulation. However, EA modulators are only able to operate over a small spectral range. For the realisation of dense wavelength division multiplexing (WDM), with a large number of channels, the growth of multiple SiGe layers, each with a different composition, is required so that each modulator operates at a different wavelength (the composition of a SiGe alloy determines both the bandgap energy^20^, and the lattice constant^21^). This will enable operation over a larger spectral range, therefore greatly enhancing the aggregate data rate of the transceiver system. Optical leakage into the Si substrate can be prevented by localised buried SiO_2_ layers, which are formed as part of the rapid melt growth (RMG) process used in this paper for SGOI formation.

This RMG technique, also referred to as liquid phase epitaxy (LPE)^22^, involves the lateral liquid phase regrowth of SiGe structures upon an insulator, from a Si seed, as shown in Fig. 1. It is enabled by the fact that Si has a much higher melting point than Ge (1414 °C and 938 °C respectively), meaning that the Ge can be melted whilst the Si, acting as a template, maintains its crystal structure. One of the major advantages of this technique is that the initial growth of Ge is non-critical because the material will be subsequently melted, meaning finely tuned growth conditions are not required. All threading dislocations, caused by the lattice mismatch between Si and Ge, are confined to the seed area and do not propagate along the SiGe structures, resulting in single crystal, device grade material^23^. Due to the high diffusivity of Si (from the substrate) in liquid Ge^24^, a whole range of SiGe compositions can be achieved from an initial pure Ge growth. Ge diffusion into the Si substrate is also observed during the melting phase^23^. Other fabrication techniques for producing SGOI include layer transfer using a donor wafer^25^ and Ge condensation^26^. However, using these techniques, only a single SiGe composition can be achieved per wafer. In previous work, we have demonstrated the ability to form uniform composition SiGe-on-insulator by RMG using tailored structures^23^. In this paper, we investigate the structural parameters that affect the SiGe composition profile, and demonstrate for the first time the ability to grow multiple uniform composition SiGe strips, each with a different composition, on a single wafer, at the same time.

## Results & Discussion

The regrowth of a simple straight SiGe strip using RMG results in a graded composition profile, with higher Si composition near the seed, and pure Ge at the distal end of the strip. This type of structure can be utilised for the growth of pure Ge for photodetectors^27^. The graded composition is a result of preferential Si rich solid formation, with rejection of Ge into the liquid, as the epitaxial regrowth front propagates along the strip, caused by the large separation between the solidus and liquidus curves of the SiGe phase diagram (Fig. 2). This type of SiGe composition profile occurs under conditions of slow regrowth front propagation velocity, therefore enabling the rejected Ge to fully diffuse into the bulk melt. In order to fabricate device grade SiGe, a uniform composition is required. This can be achieved with the addition of radiating branches, added to the main SiGe strip, as detailed in Fig. 3. These branches increase the cooling rate of the structure, and therefore also increase the regrowth front propagation velocity, so that any rejected Ge at the regrowth front is incorporated into the solid before it can diffuse into the bulk melt. Figure 4 details the effect of the branch dimensions on the composition profile in the main central strip of the tree-like structures. The thickness of the SiGe layer is 400 nm.

Figure 4a shows a range of branch separations with a fixed branch width (5 μm) and branch length (20 μm). The main strip is 100 μm long in all cases. The branch separation is reduced by adding additional branches ranging from *n* = 4 (*B*_*S*_ = 25 μm) to *n* = 10 (*B*_*S*_ = 5 μm). There is a clear change in the composition profile of these structures when compared with the straight strip (no branches). This is attributed to the cooling effects of the branches, which means that there is no longer complete mixing of the rejected Ge into the liquid ahead of the regrowth front, due to a faster regrowth front propagation velocity. Therefore, a more uniform composition profile is achieved, until the end of the strip where the remaining distance left to solidify is short enough that complete mixing of the rejected Ge into the liquid occurs. It can be seen that there is negligible change in the composition profile when *B*_*S*_ < 10 μm, meaning that the cooling rate capability of the branches must saturate at this point.

Figure 4b shows a range of branch widths with a fixed branch length (20 μm). The branch separation varies from 3 μm for the widest branch (*B*_*W*_ = 5 μm) to 7 μm for the narrowest branch (*B*_*W*_ = 1 μm) in order to keep the total length of the tree-like structure constant (*C*_*L*_ = 65 μm). This small variation in *B*_*S*_ has been shown in Fig. 4a to have very little effect on the composition profile, so for the purposes of this discussion it will be neglected. It can be seen that whilst increasing *B*_*W*_ leads to a slight flattening of the composition profile, the effect is much less profound than increasing *B*_*L*_ by the same proportion (i.e. *B*_*L*_ = 5 μm to 25 μm and *B*_*W*_ = 1 μm to 5 μm), as shown in Fig. 4c which shows a range of branch lengths with a fixed branch width (5 μm) and branch separation (3 μm). As *B*_*L*_ is extended the composition profile becomes more uniform. This shows that extending *B*_*L*_ increases the cooling rate much more effectively than extending *B*_*W*_. In order to demonstrate this, Fig. 4d plots three different branch dimensions, each with the same total volume (8 μm^3^). The flatter composition profiles caused by the long, thin branches indicate a faster regrowth front propagation velocity, and therefore an increased cooling rate. The long, thin branches have an increased surface area exposed to the SiO_2_ (due to the increased sidewall surface area of the 3D structures) when compared with the short, wide branches. We can then hypothesize that when a larger surface area is exposed to the SiO_2_ it leads to increased heat dissipation (the exposed sidewall surface areas are 16.4 μm^2^, 8.8 μm^2^, and 5.6 μm^2^ for the 20 × 1 × 0.4 μm^3^ branches, 10 × 2 × 0.4 μm^3^ branches, and 5 × 4 × 0.4 μm^3^ branches, respectively).

For these particular structural dimensions the composition profile does not become uniform for *B*_*L*_ ≤ 25 μm. However, in previous work we have observed a uniform composition profile with structural parameters *C*_*W*_ = 3 μm, *B*_*W*_ = 5 μm, *B*_*L*_ = 20 μm, *B*_*S*_ = 3 μm and *C*_*L*_ = 65 μm^23^ (shown in Fig. 4c), the only difference being a decrease in *C*_*W*_. This suggests that in order to achieve a uniform composition profile in the central strip, the branches must be wider than the central strip. The maximum width however, is limited to 5 μm as SiGe agglomeration is observed in the melt at greater widths^28^. This therefore limits the central strip width t_o_ <5 μm using this particular fabrication process.

To understand how the cooling rate affects the composition profile in the central strip of the tree-like structures the segregation coefficient, *k*, must be considered. At any given temperature, *k* can be defined as the ratio between the Ge composition in the solid, *C*_*S*_, and the Ge composition in the liquid, *C*_*L*_, at the regrowth interface, which can be calculated from the SiGe phase diagram^29^:


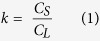


This equation is only correct under equilibrium conditions, which is approximately true when the regrowth front propagation velocity is slow. However, under non-equilibrium conditions (i.e. when the regrowth front propagation velocity is fast, as with the tree-like structures) the Ge composition is non-uniform in the melt ahead of the regrowth front. Consequently, the effective segregation coefficient, *k*_*e*_, must be considered instead^30^:


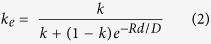


where *R* is the regrowth front propagation velocity, *D* is the diffusion coefficient of Ge in liquid SiGe^31^, and *d* is the length of the boundary region with non-uniform Ge distribution. From this equation it can be seen that as *R* increases, *k*_*e*_ tends towards unity. In practice, this implies that a high regrowth front propagation velocity will lead to minimal segregation at the regrowth front, and therefore a uniform composition profile.

Most crucially, in order to grow multiple uniform SiGe compositions on the same wafer, the tree-like structures described above have been combined with a straight strip, to incorporate the graded composition profile of the straight strip with the uniform composition profile of the central strip of the tree-like structures. This novel structure results in a range of tuneable uniform compositions, as shown in Fig. 5.

This structure demonstrates a Ge composition of 81%, 92%, 95%, and 97% in the central strip of the first, second, third, and fourth tree-like structures, respectively. The standard deviations of the 7 measured points in each tree-like structure are 0.006, 0.008, 0.004, and 0.002, respectively, showing that the composition can be controlled to within 1% of the mean (which is within the measurement error), over a 56 μm length, using this method. This length could potentially be extended beyond 56 μm simply by modifying the structural design by increasing the central strip length and adding more branches. An investigation is currently underway to determine the maximum achievable length. Lower Ge compositions could also be achieved with a higher anneal temperature, as per the SiGe phase diagram. This design has the potential to be extremely powerful for SGOI growth as it enables a multitude of uniform compositions to be achieved using a single growth step, therefore allowing a large degree of design freedom across a single wafer.

In conclusion, we have demonstrated the ability to locally tune the SiGe alloy composition by means of an RMG technique with tailored structures for high quality SGOI layer formation, using only a single Ge growth step and a single anneal step. This is revolutionary for applications that require multiple crystalline SiGe compositions on the same substrate, such as wavelength division multiplexing (WDM) systems. The composition is dictated by structural design, and not by the deposition or growth method. Furthermore, the layers are grown using simple, CMOS compatible, low cost fabrication methods, with a low thermal budget. This technology could pave the way for the seamless integration of electronics and photonics on a variety of substrates such as bulk Si or SOI.

## Methods

### Fabrication process

**Figure 1:**
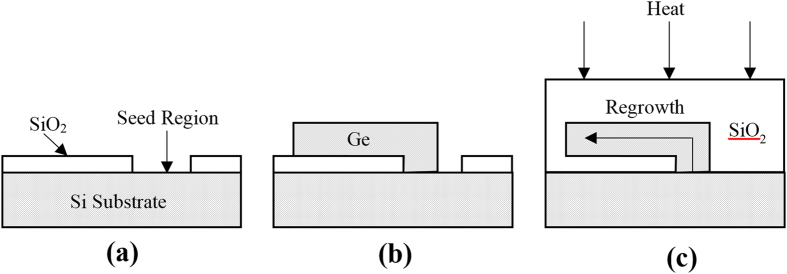
RMG fabrication process summary (cross-section view). (**a**) SiO_2_ deposition and patterning, (**b**) Ge growth and patterning, (**c**) encapsulation and rapid melting/cooling. Si diffuses from the substrate into the liquid Ge during the melting phase, resulting in solid SiGe formation during cooling.

**Figure 2:**
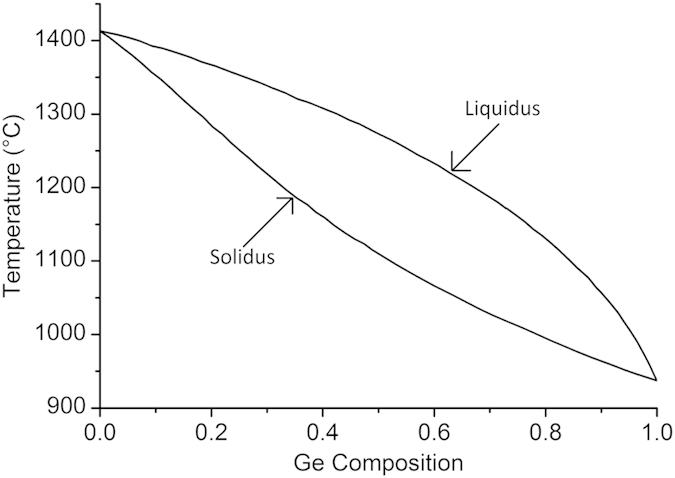
SiGe phase diagram – adapted from^29^.

**Figure 3:**
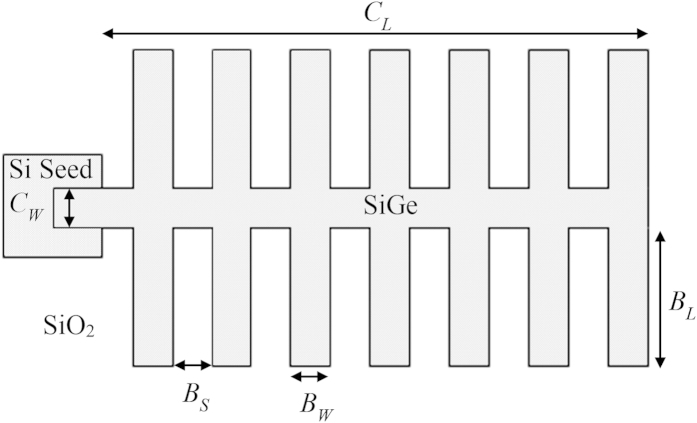
Schematic of a tree-like structure (plan view). Dimensions are defined as: centre strip width = *C*_*W*_, centre strip length = *C*_*L*_, branch width = *B*_*W*_, branch length = *B*_*L*_, branch separation = *B*_*S*_, and number of branches = *n*.

**Figure 4:**
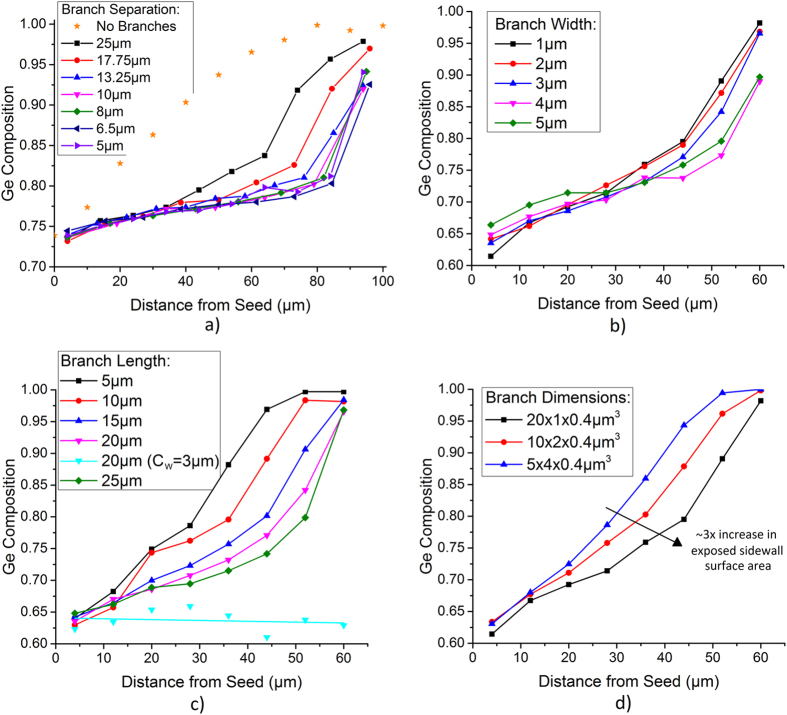
Ge composition as a function of distance from the Si seed for the central strip of tree-like structures with a range of branch dimensions. (**a**) *C*_*W*_ = 5 μm, *B*_*W*_ = 5 μm, *B*_*L*_ = 20 μm and *C*_*L*_ = 100 μm. The total number of branches increases from n = 4 (*B*_*S*_ = 25 μm) to n = 10 (*B*_*S*_ = 5 μm). Estimated maximum SiGe temperature = 1030 °C, (**b**) *C*_*W*_ = 5 μm, *C*_*L*_ = 65 μm and *B*_*L*_ = 20 μm. *B*_*S*_ = 3 μm to 7 μm, resulting in equal number of branches on each structure (*n* = 8), (**c**) *C*_*W*_ = 5 μm (or *C*_*W*_ = 3 μm^23^), *B*_*W*_ = 5 μm, *B*_*S*_ = 3 μm, *C*_*L*_ = 65 μm and n = 8, (**d**) *C*_*W*_ = 5 μm, *C*_*L*_ = 65 μm and n = 8. Estimated maximum SiGe temperature for (**b**–**d**) = 1055 °C.

**Figure 5:**
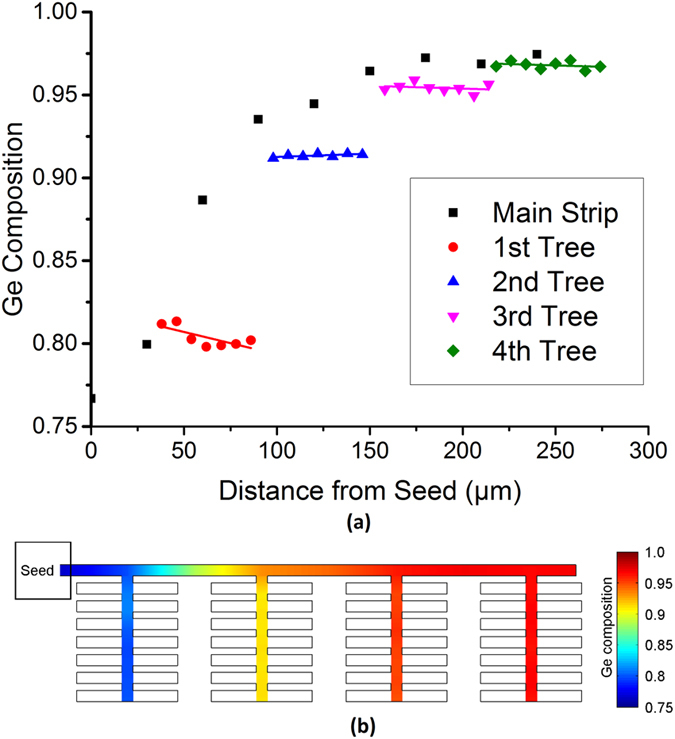
Ge composition as a function of distance from the Si seed for a straight strip with a series of attached tree-like structures. Tree properties: *C*_*W*_ = 3 μm, *B*_*W*_ = 5 μm, *B*_*L*_ = 20 μm, *B*_*S*_ = 3 μm, *C*_*L*_ = 65 μm and n = 8. Main strip dimensions: width = 5 μm and length = 230 μm. Trees are located at distances of 30 μm, 90 μm, 150 μm and 210 μm from the Si seed. Estimated maximum SiGe temperature = 1017 °C. The final pair of branches from each tree-like structure have been excluded from the plots as the composition becomes non-uniform in these regions. The white regions in (**b**) have not been measured as in practice they would be etched away, leaving only the uniform composition SiGe. The central strip of each tree-like structure has a linear fit applied.

All fabrication was carried out using 6 inch <100> bulk Si wafers. Firstly, a 50 nm SiO_2_ layer was deposited using plasma enhanced chemical vapour deposition (PECVD). This SiO_2_ layer was patterned using standard UV photolithography and a dilute (20:1) HF etch in order to expose the underlying Si, which acts as a seed for the SiGe regrowth process. A 400 nm Ge layer was then grown using a non-selective PECVD process. This was patterned into the desired structures using standard UV photolithography and an inductively coupled plasma (ICP) etch, to leave Ge structures emanating from the Si seeds. A 1 μm SiO_2_ layer was subsequently deposited by PECVD in order to encapsulate the Ge structures, forming micro-crucibles. Finally, the samples were heated in a rapid thermal annealer (RTA) in order to melt the encapsulated Ge and initiate recrystallization upon cooling. The temperature within the RTA chamber was stabilized at 500 °C, before ramping to the maximum temperature (in the range 1030 °C to 1055 °C) at a rate of approximately 100 °C/s. The wafers were held at the maximum temperature for 1 second before cooling naturally. The cold walls of the RTA chamber assist in the rapid cooling process. This process is summarized in Fig. 1.

### Material characterization

The SiGe composition in the central strip of the tree-like structures has been characterized using 532 nm Raman spectroscopy, with a 50x objective lens, which results in a laser spot size of approximately 0.5 μm. The penetration depth of the laser, which is dependent on the SiGe composition, is in the range 17 nm to 83 nm for the compositions measured^32^. The Ge composition, *x*, has been calculated using the Mooney equation^33^, with a *k* value of 1.2^23^, by measuring the ratio of the SiGe mode intensity (at approximately 380 cm^–1^) to the GeGe mode intensity (at approximately 300 cm^–1^) at each point in a lateral scan of the central strip. The SiGe composition is then plotted as a function of the distance from the Si seed.

## Additional Information

**Data Availability**: The data for this paper can be found at 10.5258/SOTON/384020.

**How to cite this article**: Littlejohns, C. G. *et al.* Towards a fully functional integrated photonic-electronic platform via a single SiGe growth step. *Sci. Rep.* **6**, 19425; doi: 10.1038/srep19425 (2016).

## References

[b1] Jalali B. & Fathpour S. Silicon photonics. J. Lightwave Technol. 24, 4600–4615 (2006).

[b2] Gardes F. Y. *et al.* Micrometer size polarization independent depletion-type photonic modulator in silicon on insulator. Opt. Express 15, 5879–5884 (2007). 19532847 10.1364/oe.15.005879

[b3] Brimont A. *et al.* Slow-light-enhanced silicon optical modulators under low-drive-voltage operation. IEEE Photon. J. 4, 1306–1315 (2012).

[b4] Hu Y., Gardes F. Y., Thomson D. J., Mashanovich G. Z. & Reed G. T. Coarse wavelength division (de) multiplexer using an interleaved angled multimode interferometer structure. Appl. Phys. Lett. 102, 251116 (2013).

[b5] Thomson D. J. *et al.* High performance mach-zehnder-based silicon optical modulators. J. Sel. Top. Quantum Electron. 19, 85–94 (2013).

[b6] Zimmermann L. *et al.* Monolithically integrated 10Gbit/sec silicon modulator with driver in 0.25μm SiGe:C BiCMOS. *IET Conference Proceedings*, 504–506 (2013).

[b7] Nedeljkovic M. *et al.* Mid-infrared thermo-optic modulators in SoI. IEEE Photon. Technol. Lett. 26, 1352–1355 (2014).

[b8] Reed G. T. *et al.* Recent breakthroughs in carrier depletion based silicon optical modulators. Nanophoton. 3, 229–245 (2014).

[b9] Duan G.-H. *et al.* Hybrid III-V on silicon lasers for photonic integrated circuits on silicon. J. Sel. Top. Quantum Electron. 20, 158–170 (2014).

[b10] Reed G. T. *et al.* Silicon photonics. Paper presented at *International Silicon-Germanium Technology and Device Meeting,* Singapore. Place of publication: 10.1109/ISTDM.2014.6874661 (2014, June).

[b11] Thomson D. J., Littlejohns C. G., Stankovic S., Nedeljkovic M. & Reynolds S. A. Silicon photonics. Encyclopedia of Electrical and Electronics Engineering (Wiley, 2015).

[b12] Xu D.-X. *et al.* Silicon photonic integration platform - have we found the sweet spot? J. Sel. Top. Quantum Electron. 20, 189–205 (2014).

[b13] Thomson D. J. *et al.* Silicon carrier depletion modulator with 10 Gbit/s driver realized in high-performance photonic BiCMOS. Laser & Photon. Rev. 8, 180–187 (2014).

[b14] Fischetti M. V. & Laux S. E. Band structure, deformation potentials, and carrier mobility in strained Si, Ge, and SiGe alloys. J. App. Phys. 80, 2234–2252 (1996).

[b15] Littlejohns C. G. *et al.* 50 Gb/s silicon photonics receiver with low insertion loss. IEEE Photon. Technol. Lett. 26, 714–717 (2014).

[b16] Littlejohns C. G. *et al.* Ge-on-Si plasma enhanced chemical vapor deposition for low cost photodetectors. IEEE Photon. J. 7, 6802408 (2015).

[b17] Liu J. *et al.* Waveguide-integrated, ultralow-energy GeSi electro-absorption modulators. Nat. Photon. 2, 433–437 (2008).

[b18] Feng D. *et al.* High speed GeSi electro-absorption modulator at 1550 nm wavelength on SOI waveguide. Opt. Express 20, 22224–22232 (2012). 23037370 10.1364/OE.20.022224

[b19] Lampin J. F., Desplanque L. & Mollot F. Detection of picosecond electrical pulses using the intrinsic Franz–Keldysh effect. App. Phys. Lett. 78, 4103–4105 (2001).

[b20] Braunstein R., Moore A. R. & Herman F. Intrinsic optical absorption in germanium-silicon alloys. Phys. Rev. 109, 695–710 (1958).

[b21] Dismukes J. P., Ekstrom L. & Paff R. J. Lattice parameter and density in germanium-silicon alloys. J. Phys. Chem. 68, 3021–3027 (1964).

[b22] Liu Y., Deal M. D. & Plummer J. D. High-quality single-crystal Ge on insulator by liquid-phase epitaxy on Si substrates. Appl. Phys. Lett. 84, 2563–2565 (2004).

[b23] Littlejohns C. *et al.* Next generation device grade silicon-germanium on insulator. Sci. Rep. 5, 8288 (2015). 25656076 10.1038/srep08288 PMC4319176

[b24] Pavlov P. V. & Dobrokhotov E. V. Self-diffusion in liquid germanium. Sov. Phys. Solid State 12, 225–226 (1970).

[b25] Taraschi G., Pitera A. J. & Fitzgerald E. A. Strained Si, SiGe, and Ge on-insulator: review of wafer bonding fabrication techniques. Solid-State Electron. 48, 1297–1305 (2004).

[b26] Tsutomu T., Naoharu S., Tomohisa M., Masamichi S. & Shin-ichi T. A novel fabrication technique of ultrathin and relaxed SiGe buffer layers with high Ge fraction for sub-100 nm strained silicon-on-insulator MOSFETs. Jpn. J. App. Phys. 40, 2866 (2001).

[b27] Na N., Tseng C.-K., Kang Y. & Lee M.-C. M. Rapid-melt-growth-based GeSi waveguide photodetectors and avalanche photodetectors. Proc. SPIE 8990, 899014 (2014).

[b28] Hashimoto T., Yoshimoto C., Hosoi T., Shimura T. & Watanabe H. Fabrication of local Ge-on-insulator structures by lateral liquid-phase epitaxy: effect of controlling interface energy between Ge and insulators on lateral epitaxial growth. Appl. Phys. Express 2, 066502 (2009).

[b29] Olesinski R. W. & Abbaschian G. J. The Ge−Si (germanium-silicon) system. Bull. of Alloy Phase Diag. 5, 180–183 (1984).

[b30] Burton J. A., Prim R. C. & Slichter W. P. The distribution of solute in crystals grown from the melt. part I. theoretical. J. Chem. Phys. 21, 1987–1991 (1953).

[b31] Brunco D. P., Thompson M. O., Hoglund D. E., Aziz M. J. & Gossmann H. J. Germanium partitioning in silicon during rapid solidification. J. Appl. Phys. 78, 1575–1582 (1995).

[b32] Humlíček J., Garriga M., Alonso M. I. & Cardona M. Optical spectra of Si_x_Ge_1−x_ alloys. J. Appl. Phys. 65, 2827–2832 (1989).

[b33] Mooney P. M., Dacol F. H., Tsang J. C. & Chu J. O. Raman scattering analysis of relaxed Ge_x_Si_1-x_ alloy layers. Appl. Phys. Lett. 62, 2069–2071 (1993).

